# Increased Levels of BAFF and APRIL Related to Human Active Pulmonary Tuberculosis

**DOI:** 10.1371/journal.pone.0038429

**Published:** 2012-06-12

**Authors:** Kai Liu, Yan Zhang, Shizong Hu, Yang Yu, Qianting Yang, Dongdong Jin, Xinchun Chen, Qi Jin, Haiying Liu

**Affiliations:** 1 MOH Key Laboratory of Systems Biology of Pathogens, Institute of Pathogen Biology, Chinese Academy of Medical Sciences & Peking Union Medical College, Beijing, People’s Republic of China; 2 Shenzhen–Hong Kong Institute of Infectious Disease, Shenzhen Third People’s Hospital, Guangdong Medical College, Shenzhen, People’s Republic of China; University of Palermo, Italy

## Abstract

**Background:**

Despite great efforts to improve diagnosis and treatment, tuberculosis (TB) remains a major health problem worldwide, especially in developing countries. Lack of concrete immune markers is still the obstacle to properly evaluate active TB. Therefore, identification of more validated biomarkers and phenotypic signatures is imperative. In particular, T cell-related biomarkers are more significant.

**Methodology:**

To understand the nature of CD4^+^ T cell-derived signatures involved in infection and disease development, we examined and analyzed whole genome expression profiles of purified CD4^+^ T cells from healthy individuals (HD), two distinct populations with latent infection (with low or high IFN-γ levels, LTB_L_/LTB_H_) and untreated TB patients. Following, we validated the expression profiles of genes in the peripheral CD4^+^ T cells from each group and examined secretion levels of distinct cytokines in serum and pleural effusion.

**Principal Findings:**

Our bio-informatic analyses indicate that the two latent populations and clinical TB patients possess distinct CD4^+^ T cell gene expression profiles. Furthermore, The mRNA and protein expression levels of B cell activating factor (BAFF), which belongs to the TNF family, and a proliferation-inducing ligand (APRIL) were markedly up-regulated at the disease stage. In particular, the dramatic enhancement of BAFF and APRIL in the pleural effusion of patients with tuberculosis pleurisy suggests that these proteins may present disease status. In addition, we found that the BAFF/APRIL system was closely related to the Th1 immune response. Our study delineates previously unreported roles of BAFF and APRIL in the development of tuberculosis, and these findings have implications for the diagnosis of the disease. Our study also identifies a number of transcriptional signatures in CD4^+^ T cells that have the potential to be utilized as diagnostic and prognostic tools to combat the tuberculosis epidemic.

## Introduction

Despite efforts to improve diagnosis and treatment, tuberculosis (TB) remains a profound burden on global health, especially in developing countries [1]. Mycobacterium Tuberculosis (*M.tb*) infection can be controlled in most infected individuals. However, in a minority of cases (5∼10%), the failure to contain the infection induces active tuberculosis [2]. Similar to the active disease, latent tuberculosis also includes a diverse range of patients [1]–[3]. This diversity makes the study of tuberculosis difficult, and more efficient tools are needed to further characterize the disease.

A well-used approach to the study of complex diseases is genome-wide transcriptional analysis. Previous studies have successfully obtained useful information regarding specific disease-associated signatures and the pathogenesis of tuberculosis using transcriptome analysis[4]–[6]. These studies have analyzed the gene expression profiles of cells involved in the immune response [5], [7], [8]. Scanning of purified peripheral blood mononuclear cells (PBMCs) rather than whole blood is also a common method [9] because it allows the identification of a set of signatures for differentiating tuberculosis patients from healthy individuals (including latent tuberculosis patients) [4], [6] and discriminating the subtle differences [5].

CD4^+^ T cell-mediated immune responses play pivotal roles in controlling the growth of *M.tb* and maintaining the homeostasis of the host [10]. IFN-γ is a cytokine that strongly promotes the T helper 1 (Th1) cell response, and it has also been demonstrated to act as a protective factor against tuberculosis in human and animal studies [10]. The IFN-γ release assay is widely used in helping to diagnose *M.tb* infection. However, the debate of its clinic use continues as IFN-γ release assay fails to distinguish between LTBI and active TB. In our study, participants with latent infection were segregated into two groups according to *M.tb*-specific IFN-γ spot-forming cells (SFCs), as determined by a well-used tool of enzyme-linked immunospot (ELISPOT) assay [11], [12]. We discovered that two latent groups (low IFN-γ expression group with lower SFCs and high IFN-γ expression group with higher SFCs) possessed distinct gene expression profiles in their purified peripheral CD4^+^ T cells. Importantly, a subset of latent individuals (with high IFN-γ expression) had transcriptional signatures similar to those of active tuberculosis patients [5], In addition, we detected an increase in the expression of BAFF and APRIL during the disease stage, but not in latency population. The strong expression levels of BAFF and APRIL in the pleural effusion of patients with tuberculosis pleurisy (TP) further indicated their effects on disease status and in extrapulmonary TB. Moreover, soluble APRIL correlated with IFN-γ expression level in the pleural effusion of patients with TP, which suggests that the correlation between APRIL and IFN-γ may be useful for predicting tuberculosis.

## Materials and Methods

### Subjects and Samples

Healthy individuals with no history of tuberculosis disease, smear positive untreated pulmonary tuberculosis patients (TB, n = 39), and patients with tuberculosis pleurisy (TP, n = 21) were recruited from the Tuberculosis hospital, China. The tuberculin skin test (TST) and a previously established *M.tb*-specific IFN-γ ELISPOT assay [11] were employed to differentiate latent tuberculosis participants (LTB, n = 68) from healthy donors (HD, n = 50). Furthermore, the participants in the LTB group were segregated into two groups according to SFC counts: LTB_L_ (n = 35, 30∼80 SFCs) and LTB_H_ (n = 33, 110∼400 SFCs). Pulmonary cancer patients (CA, n = 14) were recruited as a disease control. Clinical diagnosis of pulmonary TB and TP was based on signs and symptoms, roentgenographic findings (Chest X-ray and/or HRCT) consistent with TB and sputum bacterium examination. All participants were HIV-negative, had no autoimmune diseases and were not subjected to immunosuppressant treatments. The subjects’ characteristics are summarized in Table 1.

**Table 1:** Subject Characteristics.

GroupsCharacteristics	HD	LTB_L_	LTB_H_	TB	TP	CA
Total No.	50	35	33	39	21	14
Sex and Age						
Female	25	15	11	13	8	9
Male	25	20	22	26	13	5
Age	33.5±20	40.7±7	46.1±6	38.5±13	34.0±13.3	56.5±13.8
TST	<5 mm	≥5 mm	≥20 mm; blister	Variable	Variable	–
SFCs	0∼5	30∼80	110∼400	110∼400	30∼400	–

*Definition of abbreviations*: HD  =  healthy donors; LTB_L_  =  patients with latent tuberculosis and 30∼80 SFCs; LTB_H_  =  patients with latent tuberculosis and 110∼400 SFCs; TB  =  pulmonary tuberculosis patients; TP =  patients with tuberculosis pleurisy; CA  =  pulmonary cancer patients; Total No.  =  total number of participants in each group; TST  =  tuberculin skin test; SFCs  =  *Mycobacterium tuberculosis* (*M.tb*)-specific IFN-γ spot-forming cells.

The total number of recruited participants in each group included separated samples for microarray analysis and further validation.

PBMCs were isolated from heparinized whole blood, and CD4+ T cells were directly purified from PBMCs using magnetic beads (BD Biosciences). Total RNA was extracted from purified CD4+ T cells (CD4-RNA) and CD4+ T cell-depleted PBMCs (N-CD4-RNA). In addition, plasma and pleural effusions were also collected. This work was approval by and carried out under the guidelines of the Ethical Committee of the Shenzhen–Hong Kong Institute of Infectious Disease, Shenzhen Third People’s Hospital and written informed consent was obtained from all participants involved in this study directly and not from the next of kin or careers because all of them are adult.

### Microarray Testing

CD4-RNA samples from identical groups (HD, n = 11; LTB_L_, n = 11; LTB_H_, n = 12; TB, n = 11) were equally mixed to create the RNA pools used for the microarray tests. The hybridization protocol for the human whole-genome oligonucleotide microarray (Agilent Technologies) and the bioinformatics analysis are shown in Supporting Information S1 (Microarray Test and Bioinformatics Analysis). The data were deposited in GEO at http://www.ncbi.nlm.nih.gov/geo/query/acc.cgi?acc=GSE27882.

### Real-Time PCR Validation

CD4-RNA was used to validate the 7 genes that were significantly up-regulated in patients with tuberculosis using real-time PCR (SYBR Green method). The N-CD4-RNA levels of *BAFF* and *APRIL* were also measured to detect their cellular distribution. The mRNA values were normalized to the housekeeping gene β-actin. Details of the real-time PCR protocol and primer sequences are available in the Online Supplement (Table S1).

### Measurement of Soluble and Membrane-bound Proteins

The levels of soluble BAFF and APRIL were detected in the plasma, pleural effusion and supernatants of *M.tb* antigen-stimulated PBMCs using commercial ELISA kits (Bender MedSystems). Membrane-bound BAFF, BAFF receptor (BAFFR) and transmembrane activator and CAML interactor (TACI) on CD4^+^ T cells with/without *M.tb* antigen stimulation were detected by flow cytometry. The levels of Th1 cytokines (IFN-γ, IL-12p70 and IL-2) in the plasma and pleural effusions were determined using Luminex platform.

**Figure 1:**
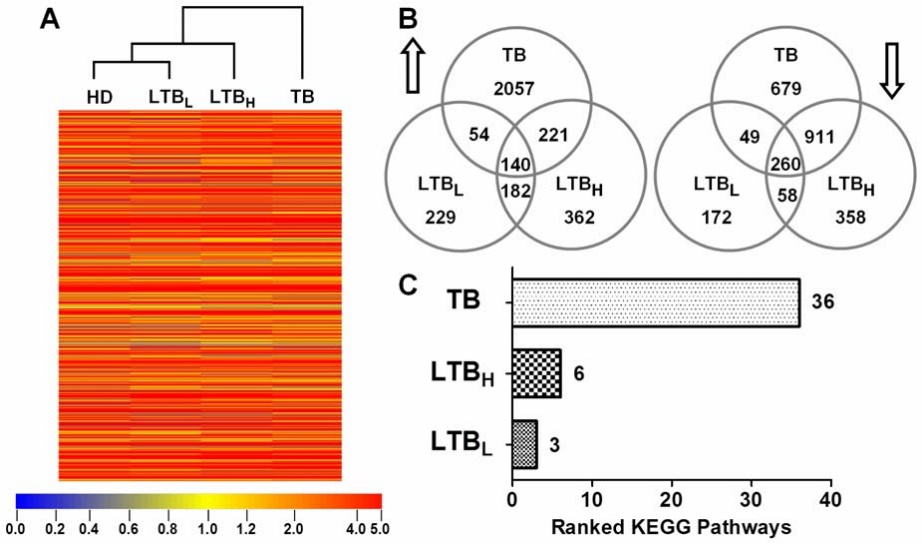
Bioinformatics analysis of gene expression in peripheral CD4^+^ T cells. A . Whole genome datasets of peripheral CD4^+^ T cells were analyzed by hierarchical clustering. The HD and LTB_L_ groups clustered directly as a new category, which was subsequently clustered with the LTB_H_ group. The TB group did not directly cluster with any group. **B**. In accordance with a 2-fold change between LTB_L_/LTB_H_/TB and HD, the number of significantly up-regulated (up arrow) and down-regulated (down arrow) genes were identified in the LTB_L_, LTB_H_ and TB groups when they were compared to the HD group. Overlapping areas represent the common significant genes shared by two or three groups. **C**. The enrichment *p* value was used to rank the KEGG pathways (*p* value <0.05, included significant gene ≥10) in the LTB_L_, LTB_H_ and TB groups. The *p* value of a KEGG pathway implies the significance of the KEGG pathway; the smaller the *p* value, the more significant the KEGG pathway. The TB group had the largest number of ranked KEGG pathways (36 pathways). The LTB_L_ and LTB_H_ groups had three and six ranked pathways, respectively.

### Data Analyses

Unpaired *t*-tests were used to analyze the difference between two groups. Pearson’s *t*-tests were employed to detect correlations between sAPRIL and the Th1 cytokines. Data are presented as the means ± SEM, and differences were considered significant if *P value* <0.05.

**Figure 2:**
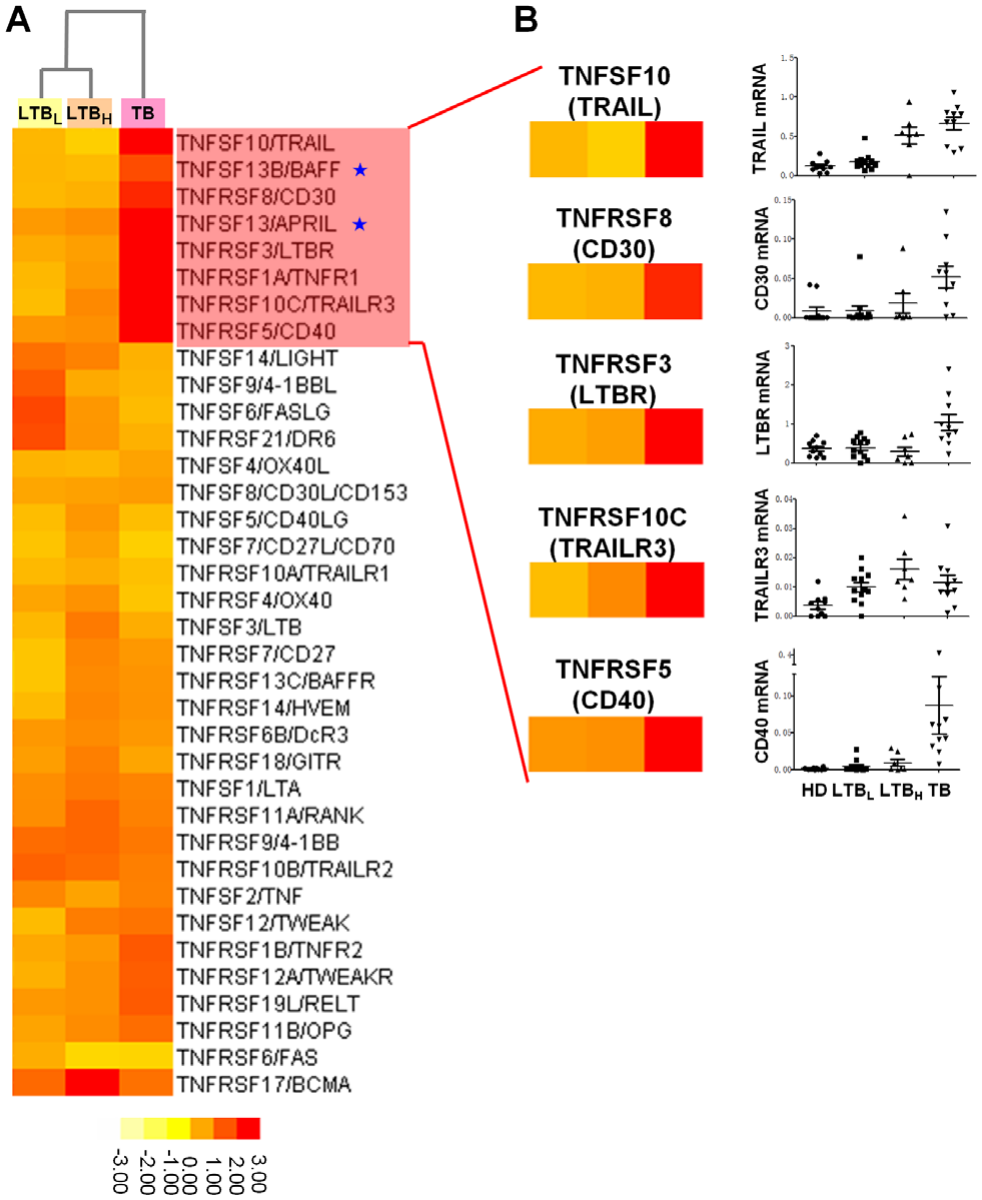
Validation of genes related to the TNF (TNF) and TNF receptor (TNFR) superfamilies (SF). **A**. Compared with the HD group, the expression levels of the genes related to the TNFSF and TNFRSF in the LTB_L_, LTB_H_ and TB groups were analyzed using a clustering algorithm. Eight genes (TRAIL, BAFF, CD30, APRIL, LTBR, TNFR1, TRAILR3 and CD40) were clustered as an identical category characterized by TB specific up-regulation. **B**. Using real-time PCR, we validated the eight TB-specific genes and detected seven genes that were significantly increased in the peripheral CD4^+^ T cells of TB patients. The mRNA values of the validated genes were normalized to the housekeeping gene β-actin. The numbers of participants included in validation test were the following: HD, n = 10; LTB_L_, n = 12; LTB_H_, n = 7; TB, n = 10. The validation results among the seven genes of the pentagram are marked BAFF and APRIL in Figure 3.A.

## Results

### Genome-wide Analysis of CD4^+^ T Cell-related Genes

Genome-wide microarray analysis was employed to examine the gene expression profiles of peripheral CD4^+^ T cells. Whole microarray datasets of CD4^+^ T cells from HD, LTB_L_, LTB_H_ and TB patients were analyzed using a clustering algorithm. The cluster analysis indicated that the two latent groups were not directly related, but the HD and LTB_L_ groups clustered to form a new category, which then clustered with the LTB_H_ group. The TB group did not cluster with any group directly (Figure 1.A). The genes that exhibited significant changes in expression (at least a 2-fold change between the HD group and other groups) were selected. Figure 1B shows the number of up- (up arrow) and down-regulated (down arrow) genes in the LTB_L_, LTB_H_ and TB groups. Compared with the HD group, the TB group possessed the largest number of significantly altered genes, followed by the LTB_H_ and LTB_L_ groups (Figure 1.B).

**Figure 3:**
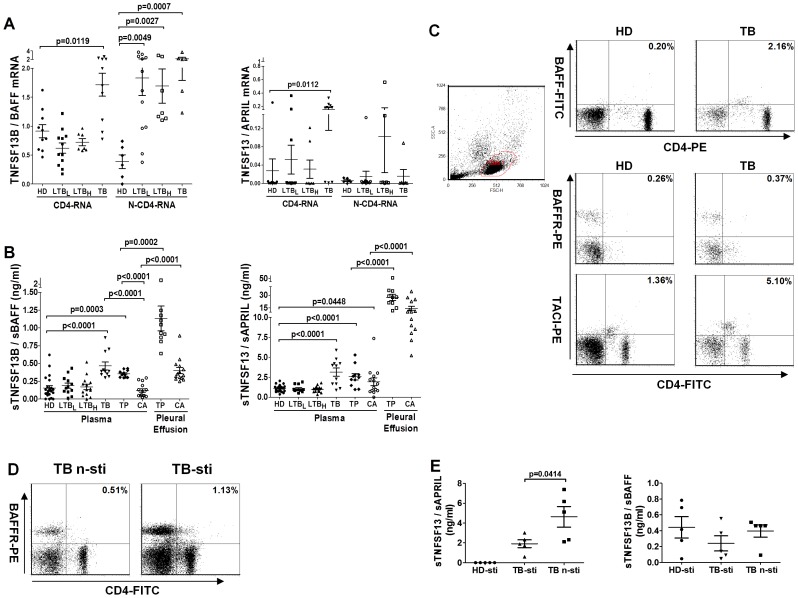
Detection of the BAFF/APRIL system-related genes. **A**. *BAFF* and *APRIL* mRNA were detected in both peripheral CD4^+^ T cells (CD4-RNA; HD, n = 10; LTB_L_, n = 12; LTB_H_, n = 7; TB, n = 10) and CD4^+^ T cell-depleted PBMCs (N-CD4-RNA; HD, n = 6; LTB_L_, n = 12; LTB_H_, n = 7; TB, n = 6). The gene expression levels of *BAFF* and *APRIL* were normalized to *β-actin*. **B**. Soluble BAFF (sBAFF) and APRIL (sAPRIL) were measured by ELISA in plasma (HD, n = 22; LTB_L_, n = 12; LTB_H_, n = 14; TB, n = 11; TP, n = 11; CA, n = 14) and pleural effusion (TP, n = 10; CA, n = 14). **C**. Flow cytometry was employed to detect BAFF, BAFFR and TACI expression on the surface of peripheral CD4^+^ T cells from HD participants (n = 2) and TB patients (n = 2). **D**. *M.tb* antigen stimulation slightly augmented the frequency of BAFFR^+^CD4^+^ T cells in PBMCs from TB patients (TB-sti, n = 2) compared with un-stimulated cells (TB n-sti, n = 2). **E**. With or without *M.tb* antigen stimulation, the levels of sBAFF and sAPRIL were detected by ELISA in the supernatants of PBMCs from TB patients (n = 5). The stimulated supernatants of PBMCs from the HD group (n = 5) were used as a control. HD-sti: *M.tb* antigen-stimulated PBMCs from HD participants; TB-sti: *M.tb* antigen-stimulated PBMCs from TB patients; TB n-sti: un-stimulated PBMCs from TB patients.

To further elucidate biological changes in the peripheral CD4^+^ T cells from the LTB_L_, LTB_H_ and TB groups, enrichment *p* values were calculated and used to rank the GO terms (*p*<0.001, included significant genes ≥20) and KEGG pathways (*p* value <0.05, included significant genes ≥10) for each group. The *p* value for each individual GO term/KEGG pathway indicates whether a term or a pathway is significant between analyzed two groups; the smaller *p* value indicates the more significance of GO term/KEGG pathway [13]. Thus, we can speculate the extent of the effects of certain genes on CD4^+^ T cells according to their GO term/KEGG pathway rankings. The LTB_L_ group had 17 ranked GO terms, which were involved in responses to stimuli, immune responses and cell-cell signaling. The LTB_H_ and TB groups had 30 and 34 ranked GO terms, respectively. Beside the GO terms ranked in the LTB_L_ analysis, significantly altered genes identified in the LTB_H_, LTB_L_, and TB groups were related to protein/RNA metabolism and protein/nucleic acid modification (Spread Sheet S1). With regard to ranked KEGG pathways (Figure 1.C), the significantly altered genes in the LTB_L_ group were annotated to include 3 ranked KEGG pathways that were mainly involved in ligand-receptor interactions and antigen processing and presentation. The LTB_H_ group contained 6 ranked KEGG Pathways, which were primarily associated with antigen processing and presentation, cell adhesion, extracellular matrix (ECM)-receptor interaction, autophagy regulation and p53 signaling. The significantly altered genes in the TB group were annotated to include 36 ranked KEGG pathways. In addition to the shared pathways of the LTB_L_ and LTB_H_ groups, the TB group-specific ranked KEGG pathways were also associated with the regulation of cellular events, such as signal transduction (in particular the MAPK signaling pathway), cytoskeletal rearrangement, adhesion, apoptosis, autophagy, and metabolism (Spread Sheet S2). These results suggest that there may be no major differences in CD4^+^ T cell function between TB and LTB_H_ patients.

**Figure 4:**
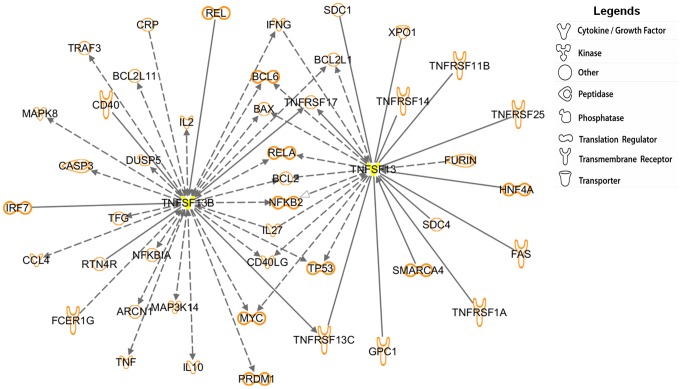
Identification of *BAFF*- and/or *APRIL*-related genes. Ingenuity Pathway Analysis (IPA) was used to identify the forty-five *BAFF-* and/or *APRIL*-related genes represented on the microarray. *BAFF* and *APRIL* (yellow marked genes) were found to interact with many genes that are involved in regulating apoptosis, transcription, the immune response and signal transduction. Among these genes, the T helper 1 (Th1)-related genes, such as IFN-γ, IL-2, IL-27 and TNF (namely, TNF-α), were identified to interact with *BAFF* and/or *APRIL*. These genes are presented by their common abbreviated names. According to their direct (solid lines) or indirect (dashed lines) action, these genes can establish contacts with *BAFF* and/or *APRIL*. Lines ending in black arrows indicate an activation pathway, and those ending in white arrows represent a translocation process. Lines joining two genes indicate binding.

### Validation of TNFSF- and TNFRSF-Associated Genes

Previous studies have reported that the TNFSF and TNFRSF genes played roles in disease of tuberculosis [10], [14]. Furthermore, we noticed that tested genes belonging to the TNF and TNF receptor superfamilies (TNFSF and TNFRSF, respectively) exhibited a significant (greater than 2-fold) change in expression levels in the array test (Table S2). Figure 2.A shows that eight genes in the TNF and TNFR superfamilies were markedly up-regulated in the TB group. Therefore, we validated the expression levels of these genes in purified peripheral CD4^+^ T cells of each group (HD, n = 10; LTB_L_, n = 12; LTB_H_, n = 7; TB, n = 10), which samples were not the same for genome array assay. Except for the expression levels of TNFR1, the expression levels of the seven other genes were confirmed by real-time PCR to be significantly increased in TB patients (Figure 2.B). The validation of BAFF and APRIL will be shown in the following section.

**Figure 5:**
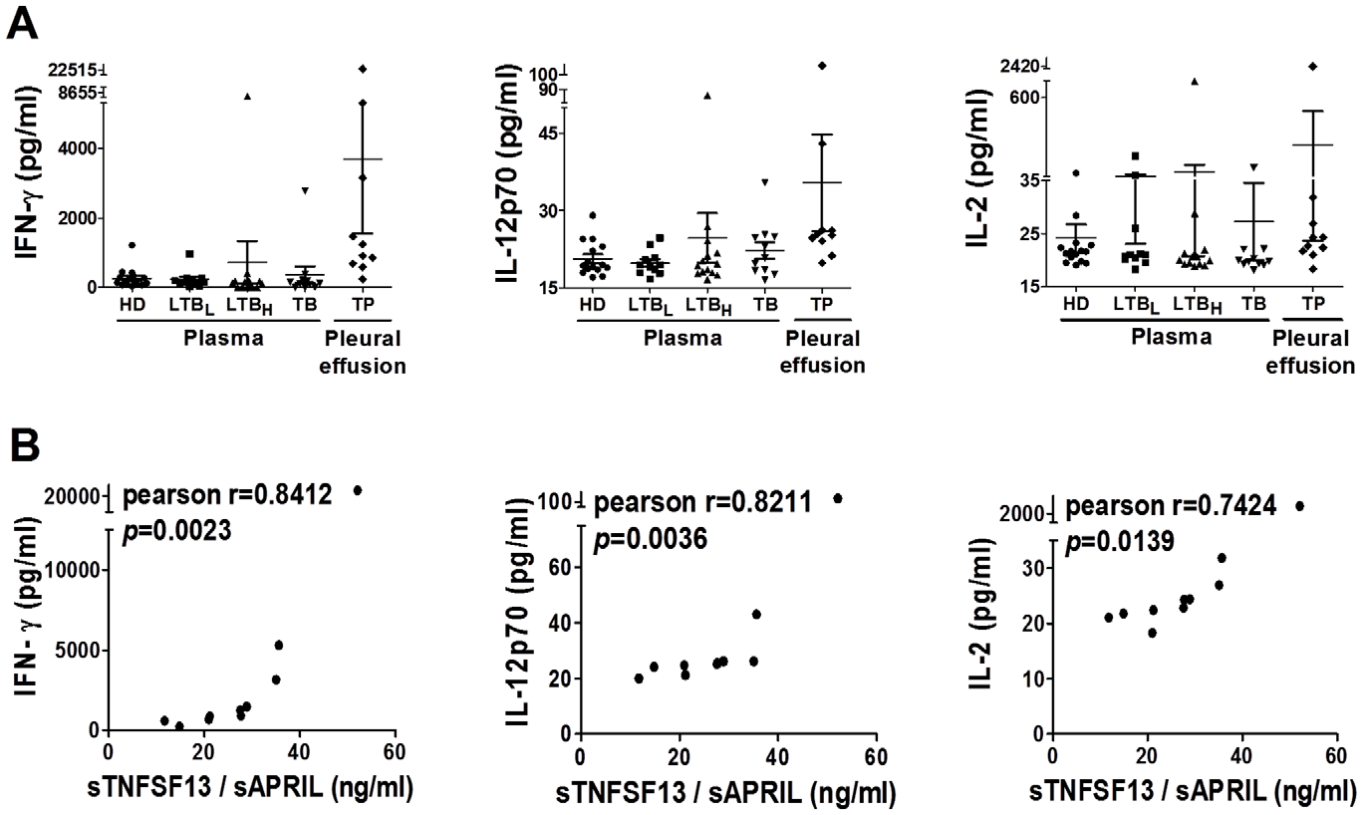
Analysis of the correlation between Th1 cytokines and sBAFF/sAPRIL. A . The levels of IFN-γ, IL-12p70 and IL-2 were detected in plasma (HD, n = 15; LTB_L_, n = 11; LTB_H_, n = 14; TB, n = 11) and pleural effusion (TP, n = 10) using Luminex technology. **B**. Significant positive correlations were identified between sAPRIL, and IFN-γ (Pearson’s correlation coefficient, *r* = 0.8412, *p* = 0.0023), IL-12p70 (Pearson’s correlation coefficient *r* = 0.8211, *p* = 0.0036) and IL-2 (Pearson’s correlation coefficient *r* = 0.7424, *p* = 0.0139) in the TP pleural effusion.

### Detection of the BAFF/APRIL System

Among the seven up-regulated genes in the group of TB, we further studied BAFF and APRIL in detail. BAFF and APRIL are ligands of the BAFF/APRIL system and expressed on many cells [15]. To examine the cellular distribution of *BAFF* and *APRIL* expression, we further analyzed the mRNA expression levels of these genes in peripheral CD4^+^ T cells and CD4^+^ T cell-depleted PBMCs. *BAFF* mRNA was significantly increased in the CD4^+^ T cells of TB patients (TB vs. HD, *p* = 0.0119). An examination of the N-CD4-RNA samples revealed that *BAFF* mRNA was significantly increased in both LTB participants (LTB_L_ vs. HD, *p* = 0.0049; LTB_H_ vs. HD, *p* = 0.0027) and TB patients (TB vs. HD, *p* = 0.0007). The significant increase in *APRIL* mRNA was only encountered in the CD4^+^ T cells of TB patients (TB vs. HD, *p* = 0.0112), and no significant up- or down-regulation could be detected in other groups (Figure 3.A). mRNA of the membrane-bound APRIL [16] ligand *TWE-PRIL* was not detected in the CD4-RNA or N-CD4-RNA samples (data not shown).

BAFF and APRIL can be secreted and act as soluble regulators [17]. Indeed, ELISA tests demonstrated that soluble BAFF (sBAFF) and soluble APRIL (sAPRIL) were significantly elevated in the plasma of TB and TP patients (Figure 3.B, TB, n = 12 vs. HD, n = 22: both sBAFF and sAPRIL, *p*<0.0001; TP, n = 10 vs. HD: sBAFF, p = 0.0003 and sAPRIL, p<0.0001). The plasma levels of sBAFF and sAPRIL were not significantly different between TB and TP patients. However, sBAFF protein levels were significantly elevated in the plasma of TB and TP patients (both were p<0.0001) compared with that of CA patients (n = 14), whereas sAPRIL levels were similar among TB, TP and CA participants. Both BAFF and APRIL showed the highest expression in the pleural effusion of TP patients, and the levels of these proteins were significantly higher than those in the pleural effusion of CA individuals. In summary, Figure 3.B shows the tuberculosis-specific up-regulation of BAFF and APRIL. In particular, the high expression of BAFF and APRIL in the TP pleural effusion was remarkable.

BAFFR and TACI are the key receptors of the BAFF/APRIL system [17]. An analysis by flow cytometry revealed that the frequencies of the CD4^+^BAFF^+^ (from 0.20% to 2.16%), CD4^+^BAFFR^+^ (from 0.26% to 0.37%) and CD4^+^TACI^+^ (from 1.36% to 5.10%) T cells were slightly increased in the PBMCs of TB patients (n = 2) compared with HD participants (n = 2) (Figure 3.C). After *M.tb*-specific antigen stimulation, the frequency of CD4^+^BAFFR^+^ T cells was enhanced in the PBMCs of TB patients (n = 2) compared with un-stimulated cells (from 0.51% to 1.13%, Figure 3.D). Interestingly, there was less sAPRIL present in the supernatants of *M.tb*-stimulated PBMCs (TB-sti) from TB patients (n = 5) than in un-stimulated PBMCs (TB n-sti) (*p* = 0.0414); furthermore, sAPRIL was not detected in the PBMC supernatants from HD patients (HD-sti, n = 5) upon *M.tb* antigen stimulation. The supernatant levels of sBAFF were not significantly different between stimulated and un-stimulated cells (Figure 3.E).

### Ingenuity Pathways Analysis For BAFF and/or APRIL-Related Genes

Ingenuity Pathways Analysis (IPA) is a common method to depict known interactions among genes [18], [19]. To further decipher possible roles of BAFF and APRIL in disease of Tuberculosis, IPA was employed to understand the BAFF- and/or APRIL-related genes represented in the microarray (Figure 4). Figure 4 shows that there are forty-five BAFF- and/or APRIL-related genes interacted, most of them are involved in regulating apoptosis, immune responses and signal transduction.

Th1 cytokines (IFN-γ, IL-2, IL-27 and TNF/TNF-α) were also identified by IPA, which suggests that there is an intimate connection between the Th1 response and the BAFF/ARPIL system. Thus, we examined the levels of Th1 cytokines (IFN-γ, IL-12p70 and IL-2) in plasma (HD, n = 15; LTB_L_, n = 11; LTB_H_, n = 14; TB, n = 11) and pleural effusion from TP patients (n = 10) by Luminex platform. IFN-γ and IL-12p20 were slightly elevated in the pleural effusion of TP patients compared with the plasma of the other groups (Figure 5.A). In the pleural effusion of TP patients, positive correlations were identified between sAPRIL and IFN-γ (Pearson’s correlation coefficient: *r* = 0.8412, *p* = 0.0023), IL-12p70 (Pearson’s correlation coefficient: *r* = 0.8211, *p* = 0.0036) and IL-2 (Pearson’s correlation coefficient: *r* = 0.7424, *p* = 0.0139) (Figure 5.B). No correlation was found between sBAFF and Th1 cytokines in TP pleural effusion.

## Discussion

In the current study, we showed that the two latent TB groups (LTB_L,_ LTB_H_) did not cluster closely. Among detected genes, LTB_L_ and HD clustered as a category, but LTB_H_ is more close to active Tuberculosis. These results suggest that LTB_L_ and HD participants have similar gene expression profiles in their peripheral CD4^+^ T cells. Although LTB_L_ and LTB_H_ are both latent TB classes, their transcription profiles are actually quite distinct. The TB group did not cluster with any other group directly, which indicates that the peripheral CD4^+^ T cells of these patients have unique gene expression profiles, and these characteristics help us to well understand TB patients from healthy and latent participants.

With regard to ranked GO terms or KEGG Pathways, the significantly altered expression of genes in the LTB_L_ group were mainly associated with stimulus responses, suggesting that the CD4^+^ T cells of the LTB_L_ participants might easily respond to *M.tb*-antigen stimulation. The CD4^+^ T cells of the LTB_H_ group could easily alternate between their roles in movement and metabolism because many migration- and metabolism-related GO terms were ranked in the LTB_H_ group. The TB group has the largest number of genes ranked by GO terms and KEGG pathways. This is reasonable to assume that the CD4^+^ T cells of TB patients do not appear to be functionally impaired and may possess the most protective potential and act for alterations in metabolism, migration, adhesion, apoptosis and respond to stimulus.

Although many studies have confirmed the indispensability of IFN-γ in the prevention of TB development, correlation of IFN-γ levels and protection ability still debate [20]. Therefore, it is essential to identify other tuberculosis-specific signatures. In this study, TNFSF and TNFRSF genes were screened for further validation because some of them have previously been confirmed to have effects on TB development. TNF-α promotes granuloma formation, which is involved in preventing the dissemination of bacilli [10], and anti-TNF-α treatment for rheumatoid arthritis increases the risk of active tuberculosis development [21]. FAS, FASLG and CD30 have also been shown to be related to disease development [14], [22], [23]. In our study, soluble CD30 and FASLG were also markedly up-regulated in the plasma of tuberculosis patients (data not shown).

Furthermore, our results indicate that BAFF and APRIL expression is significantly elevated during active tuberculosis infections. And, a stronger secretion of soluble BAFF and APRIL was observed in the pleural effusion of TP patients, which suggests that the two cytokines are involved in pulmonary and extraplumonary Tuberculosis and may reflect disease exacerbation. Although the levels of sAPRIL were lower in the *M.tb* antigen-stimulated supernatants than in un-stimulated supernatants of TB patients, we hypothesize that the down-regulation of sAPRIL is caused by its consumption during stimulation because it has been shown to play an anti-apoptotic role through an autocrine pathway [24], [25].

BAFF and APRIL previously reported as two important mediators of B cells [17], also affect the survival and activation of T cells [26]–[28]. In particular, BAFF also promotes the Th1 response [29]. Certain cytokines, such as IFN-γ, IL-10 and IFN-α, can induce various cells to augment BAFF production [30], [31]. However, IL-4 can inhibit BAFF expression [31]. Similar to BAFF, APRIL has been shown to be up-regulated in response to IFN-γ and IFN-α treatment [30], [32]. In our study, TB and TP patients secreted high antigen specific IFN-γ level detected by ELISPOT (SFCs>110), and plasma level of IFN-γ were markedly increased in the pleural effusion of TP patients but with low IL-4 level (Figure S1). Therefore, the dramatic enhancement of sBAFF and sAPRIL levels in patients might be caused by the stronger IFN-γ-mediated effects that outweigh the inhibitory effects of IL-4. The exact mechanisms of sBAFF and sAPRIL production require further examination. BAFF has two forms: membrane-bound and soluble [33]. An analysis by flow cytometry revealed that the frequency of BAFF^+^CD4^+^ T cells is increased in TB patients (Figure 3.C). Some studies have suggested that only immobilized BAFF can enhance the human T cell response to anti-CD3/TCR activation [26]–[28]. Thus, it appears that sBAFF might act as a suppressor and inhibit the binding of membrane-bound BAFF to its receptors.

Figure 4 shows there are forty-five known genes that interact with *BAFF* and/or *APRIL*. An IPA analysis was employed to analyze the interactions and RT-PCR results indicated that a largest number of pathways emanate from apoptosis-related genes, such as BCL2 (data not shown). Therefore, network-regulated apoptosis is important for the fate of CD4^+^ T cells and might indirectly affect tuberculosis outcome. As an autocrine factor [24], [25], [34], BAFF has also been reported to induce BCL2 up-regulation through BAFFR-mediated signaling [35] and to promote T cell survival [27]. The cytoplasmic tail of BAFFR has TNF receptor-associated factor (TRAF)-interacting motifs (TIMs) [36], which are involved in mediating cell survival, activation and differentiation. Thus, BAFF might prevent peripheral CD4^+^ T cells from apoptosis in patients and this mechanism might be related to the induction of *BCL2* gene expression.

Figure 4 also shows the intimate association between the Th1 response and the BAFF/APRIL system. The Th1 response plays an indispensable role in *M.tb* control and elimination [37]–[39]. In particular, sufficient macrophage activation is produced by IFN-γ and IL-12, thus permitting more efficient bacilli destruction [20]. Deficiencies in IL-12 or IFN-γ or their receptors render individuals more susceptible to mycobacterial infections [40], [41]. TNF-α is important for establishing granulomas and the localized control of *M.tb* [10]. A few publications revealed that BAFF is expressed on activated T cells and promotes the Th1 response [26], [28]. BAFF and IFN-γ have also been shown to establish an inflammatory loop between T and myeloid cells that exacerbates autoimmunity [29]. To our knowledge, no direct relation between APRIL and the Th1 response has been reported. However, among the TNFSF-related genes, APRIL is most closely related to BAFF; they share ∼30% sequence identity in their TNF homology domains (THDs) [42]. APRIL may play a similar role in disease of Tuberculosis. Moreover, we found strong positive correlations between Th1 cytokines (IFN-γ, IL-12p70 and IL-2) and sAPRIL in the pleural effusion of TP patients. Thus, these studies suggest a reciprocal relationship between the BAFF/APRIL system and the Th1 response in pulmonary and extrapulmonary TB patients. In our study, we found effects of the BAFF/APRIL system on tuberculosis to be intriguing. Future studies involving novel ideas and participants are necessary to discern the role of the BAFF/APRIL system in the pathogenesis of tuberculosis.

In summary, our results herein suggest that the elevated expression levels of BAFF and APRIL are associated with active pulmonary and extrapulmonary tuberculosis and that the BAFF/APRIL system is intimately correlated with the Th1 response. These results implicate a potential use for the combination of BAFF and/or APRIL with IFN-γ in the diagnosis of tuberculosis. Moreover, the CD4^+^ T cell-derived transcriptional signatures of distinct latency populations might be helpful to identify subsets of latent individuals that produce protective responses against tuberculosis. However, longitudinal studies must be performed to assess this hypothesis.

## Supporting Information

Figure S1 **The Levels of IFN-γ and IL-4 in Pleural Effusion from TP (n = 10) and CA (n = 5) patients.** Secretion levels of IFN-γ and IL-4 were detected in pleural effusion from TP (tuberculosis pleural, n = 10) and CA (lung cancer, n = 5) patients by Luminex.
(TIF)

Spread Sheet S1 **An excel spread sheet listing the ranked GO terms in LTB_L_, LTB_H_ and TB.**
(DOC)

Spread Sheet S2 **An excel spread sheet listing the ranked KEGG pathways in LTB_L_, LTB_H_** **and TB.**
(DOC)

Supporting Information S1 **Microarray Test and Bioinformatics Analysis.**
(DOC)

Table S1 **Target Genes and Primer Sequences.** Primer pairs of selected genes shown in Table S1. *β-actin was a housekeeping gene.
(DOC)

Table S2 **The Expression Level of TNFSF (A) and TNFRSF (B) family genes in Microarray Test.** Definition of abbreviations: HD = healthy donors; LTB_L_ = latent tuberculosis participants with low SFCs; LTB_H_ =  latent tuberculosis participants with high SFCs; TB = pulmonary tuberculosis patients. ID REF is the ProbeName on microarray. The expression level was displayed as ratio between every two groups. The fold change ≥2 or ≤0.5 between two groups was set as cutoff to select significant genes. Gray bar indicated the existence of significant genes.
(DOC)
