# Supplementary figures and images for: Increased Levels of BAFF and APRIL Related to Human Active Pulmonary Tuberculosis
Source: PLoS One. 2012 Jun 12;7(6):e38429. doi: 10.1371/journal.pone.0038429 (PMC3373577; doi:10.1371/journal.pone.0038429)

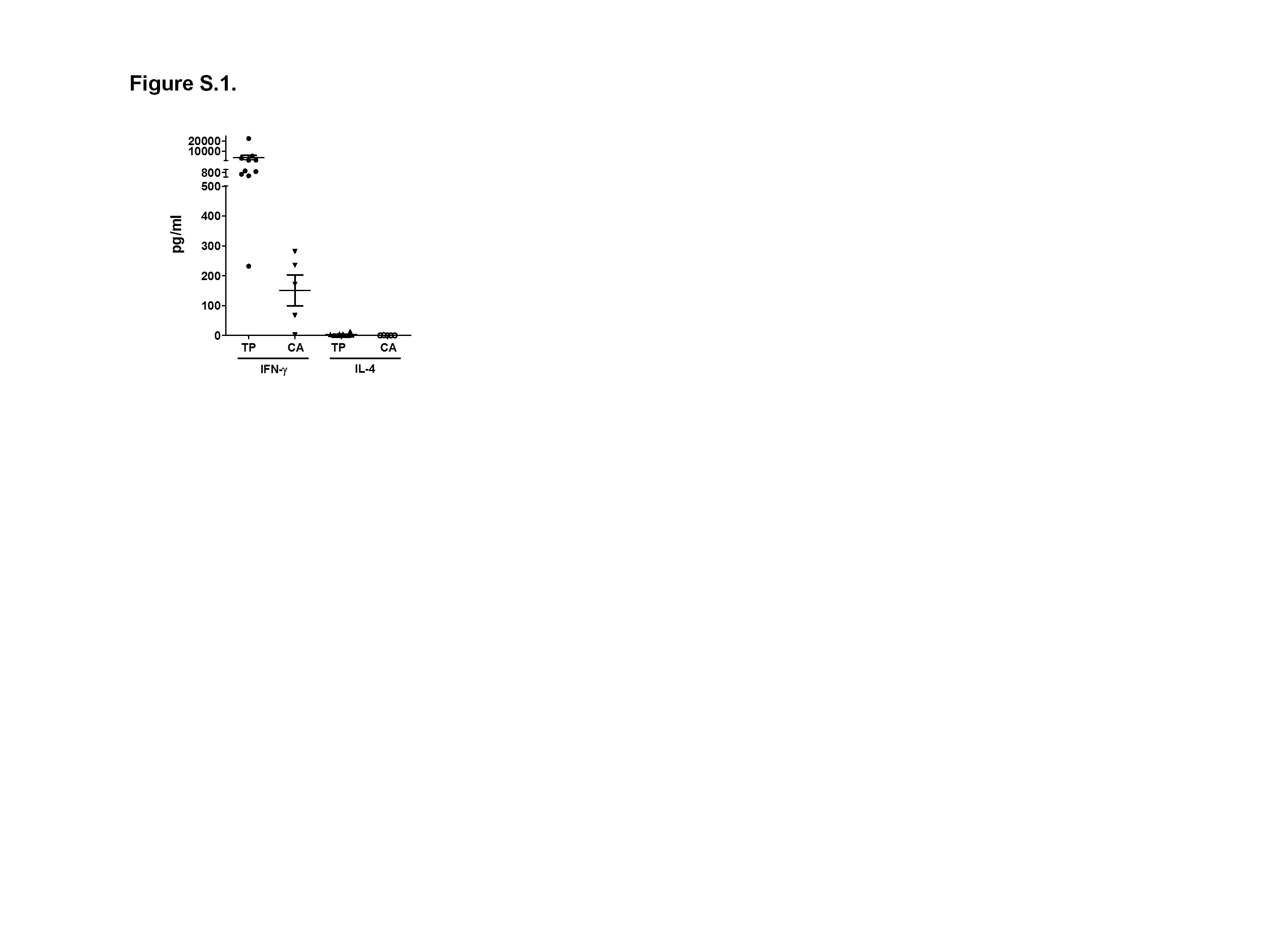

Supplement: Figure S1 — The Levels of IFN-γ and IL-4 in Pleural Effusion from TP (n = 10) and CA (n = 5) patients. Secretion levels of IFN-γ and IL-4 were detected in pleural effusion from TP (tuberculosis pleural, n = 10) and CA (lung cancer, n = 5) patients by Luminex. (TIF) [file pone.0038429.s001.tif]
