# Supplementary material for: Increased Levels of BAFF and APRIL Related to Human Active Pulmonary Tuberculosis
Source: PLoS One. 2012 Jun 12;7(6):e38429. doi: 10.1371/journal.pone.0038429 (PMC3373577; doi:10.1371/journal.pone.0038429)
Supplement: Spread Sheet S1 — An excel spread sheet listing the ranked GO terms in LTBL, LTBH and TB. (DOC) [file pone.0038429.s002.doc]

**Spread Sheet S.1. Ranked GO terms in LTB_L_, LTB_H_** and TB

| **GO terms** | **Significant genes (≥20)** | **p Value (<0.001)** |
| --- | --- | --- |
| ***biological_process of LTB_L_* (n=17)** |  |  |
| GO:50896: response to stimulus | 144 | 8.21E-08 |
| GO:50874: organismal physiological process | 134 | 4.93E-07 |
| GO:6950: response to stress | 87 | 1.43E-07 |
| GO:9607: response to biotic stimulus | 80 | 1.36E-07 |
| GO:6952: defense response | 78 | 8.02E-08 |
| GO:6955: immune response | 72 | 9.87E-08 |
| GO:9605: response to external stimulus | 65 | 7.30E-07 |
| GO:43207: response to external biotic stimulus | 55 | 4.29E-08 |
| GO:7186: G-protein coupled receptor protein signaling pathway | 55 | 8.47E-05 |
| GO:9613: response to pest, pathogen or parasite | 53 | 5.07E-08 |
| GO:9628: response to abiotic stimulus | 52 | 5.43E-10 |
| GO:42221: response to chemical stimulus | 48 | 1.94E-10 |
| GO:9611: response to wounding | 43 | 1.83E-07 |
| GO:7267: cell-cell signaling | 42 | 0.000569 |
| GO:6954: inflammatory response | 27 | 1.63E-06 |
| GO:42330: taxis | 21 | 3.88E-07 |
| GO:6935: chemotaxis | 21 | 3.88E-07 |
|  |  |  |
| ***biological_process of LTB_H_* (n=30)** |  |  |
| GO:7582: physiological process | 1335 | 0.000313 |
| GO:44238: primary metabolism | 876 | 0.000137 |
| GO:43170: macromolecule metabolism | 565 | 0.000983 |
| GO:6139: nucleobase, nucleoside, nucleotide and nucleic acid metabolism | 457 | 4.12E-06 |
| GO:6351: transcription, DNA-dependent | 281 | 0.000981 |
| GO:43412: biopolymer modification | 254 | 8.38E-05 |
| GO:6464: protein modification | 245 | 0.000204 |
| GO:6950: response to stress | 149 | 6.92E-05 |
| GO:9607: response to biotic stimulus | 130 | 0.000491 |
| GO:6952: defense response | 127 | 0.000216 |
| GO:6955: immune response | 119 | 6.51E-05 |
| GO:6512: ubiquitin cycle | 104 | 0.000148 |
| GO:16070: RNA metabolism | 94 | 1.53E-06 |
| GO:6396: RNA processing | 82 | 1.28E-06 |
| GO:9628: response to abiotic stimulus | 68 | 0.000471 |
| GO:42221: response to chemical stimulus | 62 | 0.000145 |
| GO:16071: mRNA metabolism | 51 | 0.000381 |
| GO:43285: biopolymer catabolism | 49 | 4.04E-06 |
| GO:8380: RNA splicing | 47 | 5.94E-07 |
| GO:6457: protein folding | 47 | 0.000438 |
| GO:30163: protein catabolism | 46 | 8.43E-06 |
| GO:6397: mRNA processing | 45 | 0.000923 |
| GO:44257: cellular protein catabolism | 40 | 2.22E-05 |
| GO:51603: proteolysis during cellular protein catabolism | 40 | 2.22E-05 |
| GO:19941: modification-dependent protein catabolism | 36 | 3.93E-06 |
| GO:6511: ubiquitin-dependent protein catabolism | 36 | 3.93E-06 |
| GO:398: nuclear mRNA splicing, via spliceosome | 29 | 0.000445 |
| GO:375: RNA splicing, via transesterification reactions | 29 | 0.000445 |
| GO:377: RNA splicing, via transesterification reactions with bulged adenosine as nucleophile | 29 | 0.000445 |
| GO:6968: cellular defense response | 21 | 0.000736 |
|  |  |  |
| ***biological_process of pulmonary TB*(n=34)** |  |  |
| GO:43283: biopolymer metabolism | 710 | 0.000319 |
| GO:50896: response to stimulus | 527 | 3.19E-11 |
| GO:43412: biopolymer modification | 455 | 0.000496 |
| GO:6464: protein modification | 441 | 0.000869 |
| GO:9607: response to biotic stimulus | 330 | 8.90E-26 |
| GO:6952: defense response | 312 | 1.03E-23 |
| GO:6950: response to stress | 291 | 8.08E-09 |
| GO:6955: immune response | 286 | 4.70E-23 |
| GO:9605: response to external stimulus | 211 | 7.75E-08 |
| GO:43207: response to external biotic stimulus | 181 | 8.11E-13 |
| GO:9613: response to pest, pathogen or parasite | 175 | 4.54E-13 |
| GO:9056: catabolism | 164 | 1.58E-05 |
| GO:44248: cellular catabolism | 147 | 6.02E-06 |
| GO:9057: macromolecule catabolism | 112 | 5.46E-06 |
| GO:44265: cellular macromolecule catabolism | 104 | 5.77E-06 |
| GO:7243: protein kinase cascade | 94 | 0.000826 |
| GO:43285: biopolymer catabolism | 76 | 3.35E-05 |
| GO:30163: protein catabolism | 74 | 1.25E-05 |
| GO:44257: cellular protein catabolism | 65 | 1.77E-05 |
| GO:51603: proteolysis during cellular protein catabolism | 65 | 1.77E-05 |
| GO:6954: inflammatory response | 64 | 0.000293 |
| GO:6959: humoral immune response | 60 | 2.16E-07 |
| GO:6643: membrane lipid metabolism | 49 | 0.000486 |
| GO:16064: humoral defense mechanism (sensu Vertebrata) | 45 | 7.36E-06 |
| GO:7249: I-kappaB kinase/NF-kappaB cascade | 45 | 0.00107 |
| GO:9967: positive regulation of signal transduction | 42 | 0.000261 |
| GO:43123: positive regulation of I-kappaB kinase/NF-kappaB cascade | 39 | 0.000105 |
| GO:43122: regulation of I-kappaB kinase/NF-kappaB cascade | 39 | 0.000337 |
| GO:19882: antigen presentation | 36 | 3.56E-09 |
| GO:19735: antimicrobial humoral response (sensu Vertebrata) | 36 | 5.52E-06 |
| GO:19730: antimicrobial humoral response | 36 | 8.39E-06 |
| GO:30333: antigen processing | 28 | 4.32E-07 |
| GO:19884: antigen presentation, exogenous antigen | 23 | 2.43E-13 |
| GO:19886: antigen processing, exogenous antigen via MHC class II | 23 | 1.85E-12 |
