# Supplementary material for: Increased Levels of BAFF and APRIL Related to Human Active Pulmonary Tuberculosis
Source: PLoS One. 2012 Jun 12;7(6):e38429. doi: 10.1371/journal.pone.0038429 (PMC3373577; doi:10.1371/journal.pone.0038429)
Supplement: Spread Sheet S2 — An excel spread sheet listing the ranked KEGG pathways in LTBL, LTBH and TB. (DOC) [file pone.0038429.s003.doc]

**Spread Sheet S.2. Ranked KEGG pathways in LTB_L_, LTB_H_** and TB

| **KEGG Pathways** | **Significant genes (≥10)** | **p value (p<0.05)** |
| --- | --- | --- |
| ***Pathways in LTB_L_* (n=3)** |  |  |
| Cytokine-cytokine receptor interaction | 25 | 3.01E-05 |
| Neuroactive ligand-receptor interaction | 17 | 0.0263 |
| Antigen processing and presentation | 10 | 0.0122 |
| ***Pathways in LTB_H_* (n=6)** |  |  |
| Antigen processing and presentation | 21 | 0.000534 |
| Cell adhesion molecules (CAMs) | 21 | 0.0343 |
| Small cell lung cancer | 19 | 0.00246 |
| ECM-receptor interaction | 17 | 0.00645 |
| Regulation of autophagy | 12 | 0.0257 |
| p53 signaling pathway | 12 | 0.0316 |
| ***Pathways in TB* (n=36)** |  |  |
| MAPK signaling pathway | 78 | 0.000264 |
| Regulation of actin cytoskeleton | 59 | 0.00113 |
| Focal adhesion | 51 | 0.0173 |
| Antigen processing and presentation | 49 | 3.40E-12 |
| Cell adhesion molecules (CAMs) | 44 | 9.36E-05 |
| Systemic lupus erythematosus | 42 | 5.10E-15 |
| Natural killer cell mediated cytotoxicity | 38 | 0.00023 |
| Hematopoietic cell lineage | 36 | 2.03E-07 |
| Insulin signaling pathway | 36 | 0.0336 |
| Leukocyte transendothelial migration | 35 | 0.00501 |
| Apoptosis | 29 | 0.000409 |
| Type I diabetes mellitus | 29 | 2.19E-08 |
| Allograft rejection | 28 | 2.28E-09 |
| Graft-versus-host disease | 28 | 6.81E-09 |
| Autoimmune thyroid disease | 27 | 4.65E-07 |
| Asthma | 25 | 1.05E-11 |
| Toll-like receptor signaling pathway | 25 | 0.00792 |
| mTOR signaling pathway | 23 | 0.0367 |
| p53 signaling pathway | 23 | 0.00128 |
| Pyrimidine metabolism | 23 | 0.0291 |
| Regulation of autophagy | 23 | 0.000829 |
| Epithelial cell signaling in Helicobacter pylori infection | 22 | 0.00292 |
| B cell receptor signaling pathway | 21 | 0.00197 |
| Long-term potentiation | 20 | 0.0365 |
| Vibrio cholerae infection | 20 | 0.00934 |
| Acute myeloid leukemia | 18 | 0.0244 |
| Glycolysis Gluconeogenesis | 17 | 0.0458 |
| Inositol phosphate metabolism | 15 | 0.0211 |
| Neurodegenerative Diseases | 15 | 0.00633 |
| Fructose and mannose metabolism | 13 | 0.0295 |
| Glutathione metabolism | 13 | 0.0134 |
| Alzheimer's disease | 12 | 0.000738 |
| Aminosugars metabolism | 12 | 0.00201 |
| Primary immunodeficiency | 12 | 0.00969 |
| Glycan structures – degradation | 11 | 0.0132 |
| Urea cycle and metabolism of amino groups | 10 | 0.0179 |
