# Supplementary material for: Increased Levels of BAFF and APRIL Related to Human Active Pulmonary Tuberculosis
Source: PLoS One. 2012 Jun 12;7(6):e38429. doi: 10.1371/journal.pone.0038429 (PMC3373577; doi:10.1371/journal.pone.0038429)
Supplement: Supporting Information S1 — Microarray Test and Bioinformatics Analysis. (DOC) [file pone.0038429.s004.doc]

**Microarray Test and Bioinformatics Analysis.**

Total RNA samples extracted from purified peripheral CD4+ T cells (CD4-RNA) of each group were mixed by equal quality to make up a RNA pool, which had 8 μg mixed RNA. Quantity and quality of RNA were verified before hybridization of the pooled RNA samples to human whole-genome oligonucleotide microarray (Agilent Technologies). Pooled RNA (1μg) of each group was amplified and labelled using Quick Amp labeling kit (Agilent) and hybridized with Agilent whole genome oligonucleotide microarray (~41,000 genes, Agilent). After hybridization and washing, microarray chips were processed and scanned by Agilent DNA microarray scanner. The resulting text files extracted from Agilent Feature Extraction Software (version 9.5.3) were imported into the Agilent GeneSpring GX software (version 7.3) for further analysis. The four microarray datasets were normalized in GeneSpring GX using the Agilent FE one-color scenario, by which we could obtain a normalized value of each gene. Microarray data was deposited in GEO at <http://www.ncbi.nlm.nih.gov/geo/query/acc.cgi?acc=GSE27882>. The cutoff used to select significant up- or down-regulated genes is the ratio of 2-fold change between two groups. GeneSpring GX software was used to perform hierarchical clustering analysis and form heat map of whole genome data. Normalized values of genes in each group experienced hierarchical clustering using Euclidean distance as a similarity metric and average linkage clustering for both genes and groups. Genes represented on the microarray were annotated by assignment to Gene Ontology (GO) Biological Process (www.ebi.ac.uk/GOA), to KEGG pathways (www.genome.jp/kegg/pathway.html) and to Ingenuity Pathway Analysis (IPA), (www.ingenuity.com). Enrichment *p value* was used to rank GO term (p value≤0.001) or KEGG

pathways (p value≤0.05) in terms of coupling to a biological condition. The *p value* for each individual GO term/KEGG Pathway, also known as the enrichment score, signifies the relative importance or significance of the GO term/KEGG Pathway among the genes in the selection compared the genes in the whole dataset. The less a *p value* is, the more a term or pathway is important or significant (2-4). Simultaneously, in this study, only ranked GO terms with significant gene ≥20 and ranked KEGG pathways with significant genes ≥10 were considered for decreasing the complexity in the process of analyzing GO terms and KEGG pathways.
