# Supplementary material for: Increased Levels of BAFF and APRIL Related to Human Active Pulmonary Tuberculosis
Source: PLoS One. 2012 Jun 12;7(6):e38429. doi: 10.1371/journal.pone.0038429 (PMC3373577; doi:10.1371/journal.pone.0038429)
Supplement: Table S1 — Target Genes and Primer Sequences. Primer pairs of selected genes shown in Table S1. *β-actin was a housekeeping gene. (DOC) [file pone.0038429.s005.doc]

**Table S.1. Target Genes and Primer Sequences**

*****β-actin was a housekeeping gene.
