# Supplementary material for: Increased Levels of BAFF and APRIL Related to Human Active Pulmonary Tuberculosis
Source: PLoS One. 2012 Jun 12;7(6):e38429. doi: 10.1371/journal.pone.0038429 (PMC3373577; doi:10.1371/journal.pone.0038429)
Supplement: Table S2 — The Expression Level of TNFSF (A) and TNFRSF (B) family genes in Microarray Test. Definition of abbreviations: HD = healthy donors; LTBL = latent tuberculosis participants with low SFCs; LTBH = latent tuberculosis participants with high SFCs; TB = pulmonary tuberculosis patients. ID REF is the ProbeName on microarray. The expression level was displayed as ratio between every two groups. The fold change ≥2 or ≤0.5 between two groups was set as cutoff to select significant genes. Gray bar indicated the existence of significant genes. (DOC) [file pone.0038429.s006.doc]

**Table S.2. The Expression Level of TNFSF (A) and TNFRSF (B) family genes in Microarray Test**

**A**

**B**
